# Artificial Intelligence and Human Psychology in Online Transaction Fraud

**DOI:** 10.3389/fpsyg.2022.947234

**Published:** 2022-10-11

**Authors:** Raheela Firdaus, Yang Xue, Li Gang, Muhammad Sibt e Ali

**Affiliations:** ^1^School of Management, North China University of Water Resources and Electric Power, Zhengzhou, China; ^2^School of Economics, Bahauddin Zakariya University-BZU, Multan, Pakistan

**Keywords:** Artificial Intelligence (AI), banks, cybercrimes, frauds, online transactions

## Abstract

Banking operations have changed due to technological advancement. On one hand, modernization in technology has facilitated the daily operation of banks; on the other hand, this has also resulted in an increase in the number of cyber-attacks. Artificial Intelligence has introduced new models to detect and prevent cybercrimes. Some fraud has also occurred due to the involvement of employees inside particular organizations. So, this study has focused on both sides: the machine as well as the human. Firstly, the research has focused on fraud diamond theory and has analyzed factors such as rationalization, capabilities, perceived pressure, and perceived opportunities to understand the psychology of the fraudster. Secondly, Artificial Intelligence characteristics, threat exposure, big data management, explainability, cost effectiveness, and risk prediction are evaluated to explore their use in fraud reduction in banks. The research data have been collected from 15 Banks in Pakistan with the help of a questionnaire using five-item Likert scales. The data have been analyzed using IBM SPSS Software. The results gained after correlation and regression analysis proved that Fraud diamond theory and AI characteristics have positive and significant effects on cybercrimes. This study is a great contribution to the banking industry of Pakistan as it provides a complete analysis to control fraud inside organizations by understanding the mindset of fraudsters with the help of fraud diamond theory. At the same time, outside fraud will be handled with the help of Artificial Intelligence. This will result in banks growth, which ultimately boosts the economy of a country.

## Introduction

Financial crime is any unsanctioned act done by a single person or group to gain monetary benefits (Europoll, 2021). The main objective behind this crime is remunerative. Examples of such crimes are tax fraud, financial statement fraud, and insurance fraud. According to the world economic forum, financial fraud is worth trillions of dollars (Hasham et al., 2019); therefore, both academia and industries are focusing on cyber crimes. According to Nicholls et al. (2021), online financial crime is where financial fraud is conducted by using computer and communication technologies to gain illegal income.

Computers and applications such as the internet have beneficial impacts on life and our ability to study and work. The internet provides smooth communication services for remote business and transactions. Remote communication also created possibilities for hackers to commit internet crimes. Internet crime or cybercrime (Wiafe et al., 2020) is defined as any crime conducted using computers or other communication tools to cause fear and anxiety, damage or harm, and destruction of property. Cybercrimes fall into two categories, namely, computer-assisted and computer-focused cybercrimes. Examples of computer-assisted cybercrimes are child pornography, fraud, money laundering, and cyber stalking, whereas examples of computer-focused cybercrimes are hacking, phishing, and website defacement. The term cybercrime is determined as any illegal act conducted with the help of a computer or internet that can cause agitation, financial damage, or destruction of property to the individual as well as financial organization like banks (Al-Khater et al., 2020).

All over the globe, banks are facing new challenges in the era of security (Acedański, 2020). In Pakistan, banks are increasing their user numbers at a brisk pace, and the resulting threats are consequently also multiplying. Financial services in Pakistan such as credit cards, online money transactions, and account information are vulnerable to theft or fabrication. During the last few years, Pakistan has faced some serious cyber breaches in the banking sector. In 2018, during a cyber-attack, the online security measures failed to prevent an attack in which overseas hackers stole customer data. Gemini Advisory, a body that provides guidance in addressing emerging cyber threats stated that the compromised records posted between January 24 and January 30 2019 are associated with Meezan Bank Limited’s internal systems being compromised. On 22 February 2019, Cyber security company “Group-IB” addressed in an advisory that money mules use fake cards to either withdraw money from ATMs or buy goods online. Debit card details belonging to 22 Pakistani banks were sold on the dark web (GEO, 2021). Despite the efforts of banks to eliminate online fraud, criminals still find ways around security measures to steal data and commit cybercrimes.

Cybercrimes are increasing with the COVID-19 Pandemic as criminals take advantage of workplace changes (Aldasoro et al., 2021). Traditional cyber security risk management systems have not kept pace with modern threats, but Artificial Intelligence offers new defenses and risk reduction. Cybercrimes include very sophisticated methods utilized by criminals such as the following: Malware, ransom ware, Distributed denial of service (DDOS) attacks, Man-in-the-middle attacks (MITM), SQL injection, Zero-day exploits, Spear-phishing, Watering hole, Web shell, Domain name systems (DNS) poisoning, Port scanning, Cross-site scripting, and Rootkits. Cybercrimes are predicted to have cost the world more than $6 trillion annually by 2021, up from $3 trillion in 2015 (Sausalito, 2020). Unfortunately, the security systems in financial organizations mostly fail to prevent these attacks. It is very difficult for the systems to support real-time monitoring of cyber risks at a big data scale as discussed by Demirbaga et al. (2019).

Computers are used by attackers to exploit a vulnerability in the system as well as by defenders to stop cybercrime. A very famous branch of computers that helps defenders to strengthen their systems is known as Artificial Intelligence (AI). Artificial Intelligence is defined as a field of computer science that can integrate computers and large data sets to solve problems and perform pattern detection. Artificial Intelligence can carry out many tasks in a manner similar to the human brain, for example, interactions, learning, analyzing, and problem solving. Artificial Intelligence can be divided into further fields such as machine learning and deep learning. Machine learning is further divided into main sub-branches like supervised learning and unsupervised learning. All of these branches and sub-branches use AI algorithms, which can learn from data sets, analyze data, and help in decision-making processes. According to IBM (2021), Artificial Intelligence is becoming a successful trend in the financial industry. It is more prominent in areas where customers deal with digital banking. Artificial Intelligence has allowed banks to maintain credit risk and has helped create in financial stability (Soni, 2019).

Furthermore, computers are the sources that both allow attackers to commit attacks and defenders counter these attacks; but it is actually the humans behind machines who commit fraud. Computers cannot hack a system without human command. Therefore, understanding human psychology is vital to stopping these crimes. For this purpose, our study uses the Fraud Diamond theory. The fraud triangle theory consists of three fundamental factors that are very important to committing fraud. These three main pillars of fraud are pressure, opportunity, and rationalization. These elements act as the backbone of fraud theory. The fraud triangle theory was analyzed by many researchers. Later the fraud triangle theory was modified by Wolfe and Hermonson. They added a fourth element to the fraud triangle theory: capability. Thus, due to both shape and the presence of four elements, it is called Fraud Diamond Theory. The newly added element capability encompasses how the fraudster must have the right designation in the organization to gain the advantage of opportunity, must have skills to exploit the network, have the confidence to achieve fraud opportunity, must deal with the nervousness that follows fraud, and finally how they must be a good fabricator (Pete Miller, 2018).

The main objective of this study is to strengthen the banking industry in Pakistan by reducing fraud. For this purpose, firstly, the study has focused on human nature and investigates factors, such as perceived pressure, perceived opportunities, rationalization, and capabilities (from the fraud diamond theory), that influence cybercrime and cause criminals to commit fraud. Secondly, the study has examined Artificial Intelligence factors such as threat exposure, control effectiveness, big data management, risk prediction, and explainability (as the general characteristics of AI) to reduce cybercrimes. This research is very beneficial for banks, especially those in Pakistan, as this is the first study addressing two important issues in the banking industry. This investigation has also extended its focus to the Pakistani banking industry (Ehsan and Javid, 2018) and has provided a better solution for banks to control not only risk but also cybercrimes with the help of its findings.

The article is arranged as follows: section “Literature review” is a discussion of the studies related to fraud diamond theory, cybercrimes, risk reduction, and Artificial Intelligence characteristics and hypothesis development; section “Methodology” data elaborates on the research methodology used in this study; section “Data, experiments, and results” is about experimentation and data handling; section “Conclusion and findings” contains a conclusion based on the experiments conducted; section “Discussion” explores detailed implications; and section “Future research” contains thoughts on future work.

## Literature Review

This section elaborates on fraud diamond theory and Artificial Intelligence characteristics used to develop the hypothesis of study based on the literature review section.

Financial Institutes should pay more attention to both their business models’ compatibility with future requirements and threats. According to the author, the major financial cybercrimes in banks due to the internet include ATM fraud, Denial of Service, and cyber-attack in cybercrime. Currently, the countermeasures used by banks are not good enough to prevent cyber-attacks (Al-Khater et al., 2020). Security is becoming a great concern to the banking industry due to the manifestation of information and communication technology, as clients place significant trust in banks. Due to transgressions in technology, many risks and threats have also appeared in this area. These risks are hacking, denial of service attacks, and violation of client privacy (Anjum and Naseem, 2013). The finance sector is a pillar by which one can measure the progress of a country. Financial firms provide assistance in almost every field—trade, business growth, farming progress, resource arrangement, credit formation, production, and transfer of money. An effective and efficient system for finance adds security to payments. One contribution from the banking sector is the use of digital money or plastic money. This is very innovative as it shifts the payment system toward a less expensive and faster system. Plastic money consists of credit cards, debit cards, ATMs, and smart cards (Chen, 2014). Due to the importance of data, companies are increasingly using Artificial Intelligence techniques.

The use of Artificial Intelligence in finance has brought about marvelous change. Luo et al. (2018) emphasized the applications of AI in finance and examined the effects of AI in finance. For organizations, the usage of information technology will be vital for the prospects and advancement in the new age. A new perspective towards crime is the capability of a fraudster, which was indicated by Wolfe and Hermanson (2004).

### Fraud Diamond Theory

This section addresses the studies related to fraud diamond theory and cybercrimes.

Modern business and life are facilitated by the application of information technology. The internet has its merits but at the same time has demerits too. The internet market is growing every day in Pakistan and cybercrimes follow in the same way. So, Pakistan needs more intelligent systems to deal with these kinds of white-collar crimes. A new problem that comes up with the rapid implementation of information technology is cybercrime. The whole society is under the influence of cybercrimes either directly or indirectly. Pakistan is still struggling to make strong laws and implement them against cybercrimes (Ullah et al., 2015). Many cybercrimes are even not discussed in the legislation of Pakistan.

Cybercrimes can be reduced by understanding the motivations for these crimes. There are many theories in this era. The four prominent theories of fraud are “Fraud scale theory,” “Fraud Pentagon Theory,” “Fraud triangle Theory,” and “Fraud Diamond Theory.” The most common and influential factors among these theories are pressure, competency, opportunity, and rationalization (Christian and Basri, 2019; Soepriyanto et al., 2021). Hence, the author of this article has selected the Diamond theory as having more important factors.

The fraud triangle theory consists of three elements: (a) perceived pressure, (b) opportunity, and (c) rationalization. Based on this theory, fraud is impossible without these three elements, and the severity of fraud depends on the strength of each element (Dorminey, 2012; Abdullahi and Monsor, 2018). The authenticity of this theory stemmed from Sutherland’s creation of the “white-collar crime” term (Wolfe and Hermanson, 2004). He provided an extension of the fraud triangle theory by adding an additional element, namely capability, so this theory was later called the fraud diamond theory. Researchers (Ruankaew, 2016; Avortri and Agbanyo, 2020) argue that although perceived pressures or incentives may exist along with opportunities and rationalizations, fraud is not possible unless the fourth element (capability) is present. In other words, potential attackers must have the skills and ability to actually commit fraud. In addition, according to Kassem (2012), Sorunke (2016), and Sujana et al. (2018), much fraud will not occur without the right people with the right ability to commit fraud. In other words, potential fraud perpetrators must have the skills and ability to commit fraud.

According to Ruankaew (2016) pressure is an important element of the fraud diamond theory. Fraud behavior usually arises from the pressure obtained by employees in an organization. Authors Howe and Malgwi (2006) and Albrecht et al. (2010) discussed how fraud behavior is caused by economic pressures such as greed, lifestyle, large expenses or personal debt, loan problems, financial losses, and inability to meet financial plans. A lot of literature studies found that who is responsible for important decision should their own wealth numbers (Broll and Wong, 2015). A person who does not care about inflation and spends a lot of money will face financial pressure. Opportunity is the second major element which enable fraudsters to commit fraud. According to Rae and Subramaniam (2008), someone will commit fraud if there is an opportunity and lack of control at their company. In addition, someone who has the opportunity will utilize their abilities to commit fraud. The third element of the fraud diamond theory is rationalization, which is a justification of an action as a natural behavior that is morally acceptable in a normal society. Jackson et al. (2010) argued that if someone perceives a particular action as fraudulent, it is impossible for them to be involved in a fraudulent action. Capability means that someone who has a position in a company is likely to commit fraud. According to Wolfe and Hermanson (2004) and Utami et al. (2019), much large-scale fraud is unlikely to occur if there are no people with special capabilities in the company. The banks experienced instability and suffered from financial crises in the period 2007–2009. Inappropriate supervision may be one cause of crises (Acedański, 2020).

### Cyber Threats and Artificial Intelligence

The literature related to cyber-attacks in the banking industry and the importance of Artificial Intelligence along with its characteristics is discussed in this section.

Information and cyber security incidents have grown rapidly both in scale and number (Fang, 2019). With 20 years of investigation and analyzing cyber-incidents, Nozominetworks (2019) considered the rapid expansion and sophistication of the recent cyber-attacks as unprecedented. There is also complete anticipation by security professionals that the cyber threats will progressively become more challenging and complex (CYFIRMA, 2021). This has compelled many organizations to introduce somewhat unpredictable and chaotic processes (CISCO, 2021). What is information and cyber security though? This question is answered by Cybersaint (2017), a set of technologies and processes responsible for protecting computer networks, associated software, and data from unauthorized access, alteration, or destruction. Protection of these computer system networks has careened into a danger zone during the last decade (Buczak and Guven, 2016; Greengard, 2016); each of the computer network security systems should have, at a minimum, an intrusion detection system (IDS), antivirus software, and firewalls. However, Sabar et al. (2018) discussed that firewalls effectively become unreliable as application programming interfaces and cloud computing string data together across different enterprises. In the context of big data from the cloud, cyber security has become a critical challenge and poses a greater risk as suggested by Talwar and Koury (2017). This is exacerbated by a lack of human capacity to screen big volumes of data for proper threat analysis (Pashazadeh and Navimipour, 2018). This prompted practitioners and researchers to look for new and better information and cyber security approaches (Sabar et al., 2018). Consequently, researchers started to investigate security solutions that employ Artificial Intelligence (AI). As the core AI subfield, machine learning is about the effective simulation of human activities as applied to speech and pattern recognition, image processing, cyber security, and even decision-making (Dragomir, 2017). Essentially, AI is about machines that simulate intelligent human behavior such as learning, thinking, and reasoning (Unnisabegum, 2019).

Big data management is a vast field and is a characteristic of the Artificial Intelligence that is used nowadays to detect precise cybercrime and intrusions (Dupont et al., 2017). Volume-able data management in recent times has been seen as a hot field by researchers all over the world (ARM, 2021). Big data analytics provides a solution because it is built to handle growing volumes of big data. Data analytics is more than capable of handling the large volumes of data organizations store (Alturki et al., 2017). The reason behind this capacity is the sophisticated data algorithms of the data analytics framework. By using big data analytics, it is possible to process, manage, and secure large volumes of data (Paek et al., 2021). Humans cannot process such volumes of data, and we must rely on AI characteristics to defend against cyber threats. Cybercrimes in the banking industry have been reported as theft is unmanageable without AI intervention to control and shape the data streaming through uncountable channels and networks (Liebergen, 2017). Big data is the art of finding specific patterns, unusual entries, and wrong entries from the surrounded network booths of the banking industry worldwide while facing a range of different threats from different operational tools. Support of big data management through AI can lead to the easy detection of threats or threat exposure. Threat exposure is another valuable characteristic of AI in the management of data and exposure of unlawful system entries with numerous optional ways of attacks (Statista, 2021). Vulnerability in the system is a key opportunity for fraudsters to exploit and commit frauds. Cybercrimes are the reasons behind blackouts in electricity, theft of defense secrets, and even the failure of weaponry in war. Confidential records such as bio industry records are stolen by cybercrimes (Galindo, 2000). Data availability can also be attacked by hackers through spoofing of important calls and with the help of denial of service attacks. These attacks are becoming more serious and are getting worse day by day (David and Solomon, 2016). Gartner explained how cyber security risks pervade every organization and are not always under the direct control of an information technology department. Increased cyber risk is real but so are the data security solutions (Kumar et al., 2021). Solutions are costly but effective; for the banking industry, the wealth of numerous customers is at stake, and securing the cost of the system through AI is cost-effective management as less human involvement and training modules are involved. Cost-effectiveness does not hinge on a lower cost being applied but on how much this cost is effective for cyber security management (Becker, 2020).

### Hypotheses of the Study

An important motivation for a person committing fraud is perceived pressure. This pressure can be caused by the society or family or an increasing inflation rate in the country. The second major motivation is perceived opportunity. This opportunity comes usually due to weak internal controls within the organization or some loopholes in the system or supervision. The opportunity for tax evasion by the taxpayer is possible because the taxpayer calculates the tax by using a self-assessment system. In this tax system, adequate supervision is required. In the absence of oversight by the tax authorities, it will create a perception of open opportunities for tax evasion. Sometimes the perpetrator feels they have done much for their organization. A person or employee who can falsely justify their wrong or illegal actions does so through rationalization. Attackers who commit cybercrimes think that they are doing rightful things or they have the right to steal things if they do not get them by the right means.

H1_0_: There is no significant relationship between the predictors of the attacker’s motivation level (perceived pressure, perceived opportunities, rationalization, and capabilities) and the cybercrimes in the banking industry of Pakistan.

Since the global financial crisis, cybercrimes in banks have gained more prominence, and there has been a constant focus on how attacks are being detected, measured, reported, and managed. Considerable research (Liebergen, 2017; Deloitte, 2021) within both academic and business circles has been concerned with the developments of cyber control methodologies to deal with current and emerging challenges. In tandem, there has been a growing influence of Artificial Intelligence in business applications, with many solutions already implemented and many more being explored. McKinsey & Co highlighted that risk functions in banks, by 2025, would need to be fundamentally different from what they are today. The broadening and deepening of regulations, evolving customer expectations, and the evolution of risk types are expected to drive the change. New products, services, and attack prevention techniques are being enabled through the application of evolving technologies and advanced analytics. Artificial Intelligence, identified as one of the technologies with important implications for risk management, can enable the building of more accurate models by identifying complex, non-linear patterns within large datasets. The predictive power of these models can grow with every bit of information added, thus enhancing predictive power over time. It is expected that Artificial Intelligence will be applied across multiple areas within a bank’s risk organization. Artificial Intelligence has also been recommended as an initiative that could help in the transformation of the attacks prevention function at banks.

H2_0_: There is no significant relationship between the predictors of defenders’ strategies (risk exposure, big data management, control effectiveness, risk prediction, and explainability) and the risk management and risk reduction of cyber threats in the banking industry of Pakistan.

## Methodology

The methodology adopted in this study is explained in the following section.

### Data Collection

Primary data are the original data collected by the scholar, particularly for the reason of originality in mind. In addition, primary data consists of first-hand information that has not been published, as the more valid primary data have not been altered or changed (David Crowther, 2012).

### Target Population

The noteworthy target populace for this study is those individuals who are working as full-time operation managers, branch managers, and AVPs in the banking sector of Pakistan in 15 banks in Karachi, Pakistan.

### Sampling Technique

The sampling procedure is classified into probability and non-probability sampling. Probability sampling is the sampling procedure in this study. A sampling includes the following: simple random sampling, simple random sampling is considered the easiest method of probability sampling, stratified random sampling, systematic random sampling, cluster (Area) random sampling, and multi-stage sampling. In this study, however, a standard sampling formula was chosen. In accordance with Saunders (2012), random sampling is more appropriate for making clusters and forming size. The researcher needs to repeat the sample collection process until the required results are achieved.

### Sample Size

The sample size of this research was 321 in Pakistan with a 99% response rate. The sample size for this study was chosen by using the general principal of pragmatism.

### Questionnaire Design

A brief presentation of the research, as well as the purpose of the study, is expressed within the cover letter of the questionnaire. There are two parts to the survey. Part (1) contains questions regarding the statistical data of respondents, comprising gender, age, and education using the Likert scale. These comprise four questions in segment (1). At that point segment (II) is with five points on a Likert scale questioning about the impact of independent variables on dependent variables, (2) comprise of 34 questions. The respondents of this study are provided with five options from the Likert scale from (1) strongly disagree, (2) disagree, (3) Neutral, (4) agree and (5) strongly agree.

### Pilot Test

A pilot test, which is also known as a pretest, is a small-scale study utilized to refine and move forward to assist the corroborative research. By conducting a pilot test, scholars will be able to guarantee that the respondents appreciate the questionnaire the scholar aims to carry out (Saunders, 2012). In this research study, a total of 50 sets of sample questionnaires are conveyed to the respondents for the pilot testing purpose.

## Data, Experiments, and Results

This section provides details of data collection, experiments conducted in this research study, and results based on experiments.

### Reliability Analysis

The data collected through the survey questionnaire is analyzed using the SPSS software program. Reliability is an assessment to ensure that the measurement is well-founded and valid. The most widely used reliability coefficient is Cronbach’s alpha, and this was employed in the current study to measure the consistency of variables in a summated scale.

Table 1 shows Cronbach’s alpha values calculated for all variables, which indicated 0.927 as the highest value produced by organizational cynicism. The values of all variables were higher than the recommended level of 0.70, indicating good reliability of variables.

**TABLE 1:** Reliability statistics fraud diamond theory.

	Cronbach’s alpha	Cronbach’s alpha based on standardized items	No. of items
Perceived pressure	0.802	0.798	5
Perceived opportunities	0.602	0.598	5
Rationalization	0.802	0.898	5
Capabilities	0.852	0.898	5
Threat exposure	0.852	0.898	5

The results elaborated on in Table 2. clearly show good values. Big data management and threat exposure have higher values as compared to other characteristics.

**TABLE 2:** Reliability statistics AI characteristic.

	Cronbach’s alpha	Cronbach’s alpha based on standardized items	No. of items
Threat exposure	0.832	0.878	5
Big data management	0.852	0.898	5
Explainability	0.752	0.798	5
Cost effectiveness	0.652	0.758	5
Risk prediction	0.772	0.688	5

### Correlation Analysis

The Pearson correlation technique was used to identify the relationship of variables with each other and whether any observed variable has perfect covariance with any other variable observed in this study.

The summarized result of the correlation is shown in the table above. The relationship between the two variables was found to be significant at a *p*-value of 0.01. This relationship shows a positive and moderate to strong relationship among variables (Table 3).

**TABLE 3:** Correlation of variables.

Banking industry			PP	PO	RZ	CB	TE	BD	CE	EX	RP
Factors	PP	Pearson correlation	1	0.274**	−0.045	0.398**	0.057	−0.012	0.142**	0.313**	0.143**
		Sig. (2-tailed)		0.000	0.389	0.000	0.281	0.813	0.007	0.000	0.006
		N	366	366	366	366	366	366	366	366	366
	PO	Pearson correlation	0.274**	1	0.294**	0.165**	0.223**	0.264**	0.298**	0.321**	0.082
		Sig. (2-tailed)	0.000		0.000	0.002	0.000	0.000	0.000	0.000	0.119
		N	366	366	366	366	366	366	366	366	366
	RZ	Pearson correlation	−0.045	0.294**	1	−0.172**	0.084	0.533**	0.120*	0.089	0.127*
		Sig. (2-tailed)	0.389	0.000		0.001	0.108	0.000	0.021	0.090	0.015
		N	366	366	366	366	366	366	366	366	366
	CB	Pearson correlation	0.398**	0.165**	−0.172**	1	0.046	−0.198**	0.250**	0.129*	−0.025
		Sig. (2-tailed)	0.000	0.002	0.001		0.382	0.000	0.000	0.013	0.632
		N	366	366	366	366	366	366	366	366	366
	TE	Pearson correlation	0.057	0.223**	0.084	0.046	1	0.105*	0.408**	0.200**	0.300**
		Sig. (2-tailed)	0.281	0.000	0.108	0.382		0.045	0.000	0.000	0.000
		N	366	366	366	366	366	366	366	366	366
	BD	Pearson correlation	−0.012	0.264**	0.533**	−0.198**	0.105*	1	0.183**	0.146**	0.316**
		Sig. (2-tailed)	0.813	0.000	0.000	0.000	0.045		0.000	0.005	0.000
		N	366	366	366	366	366	366	366	366	366
	CE	Pearson correlation	0.142**	0.298**	0.120*	0.250**	0.408**	0.183**	1	0.502**	0.305**
		Sig. (2-tailed)	0.007	0.000	0.021	0.000	0.000	0.000		0.000	0.000
		N	366	366	366	366	366	366	366	366	366
	EX	Pearson correlation	0.313**	0.321**	0.089	0.129*	0.200**	0.146**	0.502**	1	0.090
		Sig. (2-tailed)	0.000	0.000	0.090	0.013	0.000	0.005	0.000		0.087
		N	366	366	366	366	366	366	366	366	366
	RP	Pearson correlation	0.143**	0.082	0.127*	−0.025	0.300**	0.316**	0.305**	0.090	1
		Sig. (2-tailed)	0.006	0.119	0.015	0.632	0.000	0.000	0.000	0.087	
		N	366	366	366	366	366	366	366	366	366

*Correlation is significant at the 0.05 level (2-tailed).

**Correlation is significant at the 0.01 level (2-tailed).

### Regression Analysis

A regression analysis was conducted to test the relationship between the predictors of AI. Hypotheses are tested; assumptions of the analysis are again outlined.

Regression analysis is used to predict the value of cybercrime based on defender strategies and Artificial intelligence. The results shown in Table 4 clearly indicate there is a strong and positive relationship between cybercrimes and opportunity, pressure, rationalization, and capability. At the same time in the second model, the relationship is clearly interpreted between cybercrime and threat exposure, big data management, control effectiveness, explainability, and risk prediction.

**TABLE 4:** Regression dependent variable (cybercrime and risk reduction).

Model	R	R square	Adjusted R square	Std. error of the estimate	Change statistics					Durbin-Watson
					R square change	F change	df1	df2	Sig. F change	
1	0.523	0.773	0.761	1.11982	0.273	22.503	6	359	0.000	1.113

Predictors: PP, PO, RZ, and CB Dependent Variable: Cybercrime										
2	0.581	0.738	0.725	1.22960	0.338	26.092	7	358	0.000	2.296

Predictors: TE, BD, CE, EX, and RP Dependent Variable: Cybercrime										

## Conclusion and Findings

The research aims to comprehend the casual aspects that affect Artificial Intelligence upon risk assessment in a cyber-transaction where threats of cybercrime are increasing day by day. The results generated after analysis proved that opportunity, pressure, rationalization, and capability have a strong relationship with the psychology of fraudsters and characteristics of AI; Threat exposure, big data management, explainability, risk prediction, and cost effectiveness are directly related to risk management. These factors could be used as strategies to defend against the unlimited strategic possibilities of the attacker. This research is very significant for banks directly and for other people in society like bank customers who suffered from cybercrimes indirectly. Honest staff members could be rewarded and dishonesty should be punished so that no one tries to avail themselves of these kinds of opportunities. There should be strict supervision, and everyone should be held accountable for their responsibilities. People should use their skills in a positive way. At the last, banks need to implement Artificial Intelligence properly to secure transactions. Controlling these kinds of attacks will increase customer trust in banks and also prevent significant monetary loss every year resulting from such attacks. The study is also useful for researchers who want to broaden their research in the field of risk reduction and Artificial Intelligence.

## Discussion

This section addresses the theoretical and practical implications of the research.

### Theoretical Contribution

This paper analyzed the fraud diamond theory in the banking sector of Pakistan.

Firstly, this research is established on diamond fraud theory with the constructs of pressure, opportunity, rationalization, and capability. These constructs are for the first time analyzed from the perspective of fraud in the Pakistani banking industry. This research is different from the studies conducted in Pakistan over the past few years such as that of Ehsan and Javid (2018). This study is very helpful in understanding the thinking of employees and situations that are favorable to committing fraud. By controlling these factors and providing awareness by conducting seminars, appreciating honest employees, giving yearly bonuses, and increasing wages according to the designation of workload, these financial losses can be avoided.

Secondly, on the basis of previous studies, this research developed and designed a scale to measure Artificial Intelligence for risk reduction in Pakistani banks. Previous studies focused on the area of risk reduction but have not carried out empirical research investigation with Artificial Intelligence. In this study, the guidance of experts combined with the previous research studies was used to design a measurement scale for Artificial Intelligence, which is an innovative method.

### Practical Implications

This is the situation where the fraudster has all the necessary skills that can result in successful fraud. It is where the fraudster recognized the particular fraud opportunity and the ability to turn it into reality. Position, intelligence, ego, coercion, deceit, and stress, are the supporting elements of capability (Wolfe and Hermanson, 2004; Mackevicius and Giriunas, 2013), though not every person who possessed motivation, opportunities, and realization may commit fraud due to the lack of the capability to carry it out or to conceal it. We have concluded that capabilities as the constant influential factor as the person with pressure, opportunities and rationalization is not a threat unless he/she is not capable of executing the cybercrime.

The banks should implement strong and secure online transaction infrastructure, so that clients can trust banks for digital services. Financial organizations like banks should be more concerned about their reputation. Secure transactions are very important for customers as well as for banks. At the same time, banks should consider internal and external control. Firstly, if there is a balance between work and salary then there will be less pressure on employees. The government should also take strong measures to control inflation in the country so that people are not pressurized to take the wrong actions to cover their daily life expenditures. Secondly, to control opportunity, the organization should implement strong supervision and control loopholes in the system. Thirdly, rationalization can be handled if the organization introduces some psychological training and provides more rewards and appreciation to honest employees. Fourthly, the banks should try to use the capabilities of their employees in a positive way.

To reduce risks and the threat of attacks, Pakistani banks should implement new and innovative Artificial Intelligence techniques. Banks can implement AI-based security systems against cybercrimes, which leads to unlimited options for trapping the cyber criminals in technological black holes. Cost effectiveness has a positive and significant influence on risk reduction; if banks will implement AI systems their cost will be utilized in a better way as these systems are more effective. Risk prediction supports banks as risk can be detected before exploitation by the attacker.

Finally, the banks should create a supportive and comfortable environment for their workers and implement effective supervision tools. By implementing Artificial Intelligence banks will not only secure their reputation but also the economy.

### Future Research

This research has some limitations regarding its research methods. The survey questionnaire is used to collect the required data. The study has limitations such as time, energy, manpower, and the number of questionnaire samples. Future research can focus on more advanced survey methods. The sample size can also be extended to analyze more results.

There is a room for research both in artificial intelligence techniques and human psychology to prevent frauds. This study covers two main subjects of the banking industry, cybercrimes and risk reduction, using fraud diamond theory and Artificial Intelligence. Further research can be conducted by considering other fraud theories or by integrating other factors into the existing theory. Some new characteristics of Artificial Intelligence can also be addressed by future studies.

Finally, there is a limitation to the contents of this study. We only tested direct variables; the future researcher can expand the contents and measure the effect of moderating variables such as family, life, the workplace, expenditure, and income in the fraud diamond theory and some moderating variables such as technology adaptability, and the influence of Artificial Intelligence on customer and support of technology.

## Data Availability Statement

The original contributions presented in the study are included in the article/supplementary material, further inquiries can be directed to the corresponding author/s.

## Ethics Statement

The studies involving human participants were reviewed and approved by Department of research North China University of water resources and electric power, Zhengzhouo, Henan, PR China. The patients/participants provided their written informed consent to participate in this study.

## Author Contributions

RF: introduction, literature review, data collection, analysis, and results. YX: supervision, guidelines, and data analysis. LG: data collection, analysis, and results. MS: analysis and results. All authors contributed to the article and approved the submitted version.

## Conflict of Interest

The authors declare that the research was conducted in the absence of any commercial or financial relationships that could be construed as a potential conflict of interest.

## Publisher’s Note

All claims expressed in this article are solely those of the authors and do not necessarily represent those of their affiliated organizations, or those of the publisher, the editors and the reviewers. Any product that may be evaluated in this article, or claim that may be made by its manufacturer, is not guaranteed or endorsed by the publisher.
